# What Is the Impact of *Impatiens parviflora* on Diversity and Composition of Herbal Layer Communities of Temperate Forests?

**DOI:** 10.1371/journal.pone.0039571

**Published:** 2012-06-29

**Authors:** Martin Hejda

**Affiliations:** Department of Invasion Ecology, Botanical Institute of the Czech Academy of Sciences, Průhonice, Czech Republic; University of Leipzig, Germany

## Abstract

The aim was to estimate the impacts of invasive *Impatiens parviflora* on forests’ herbal layer communities. A replicated Before-After-Control-Impact field experiment and comparisons with adjacent uninvaded plots were used. The alien’s impact on species richness was tested using hierarchical generalized mixed effect models with Poisson error structure. Impact on species composition was tested using multivariate models (DCA, CCA, RDA) and Monte-Carlo permutation tests. Removal plots did not differ in native species richness from neither invaded nor adjacent uninvaded plots, both when the treatment’s main effect or its interaction with sampling time was tested (Chi^2^ = 0.4757, DF = 2, p = 0.7883; Chi^2^ = 7.229, DF = 8, p = 0.5121 respectively). On the contrary, ordination models revealed differences in the development of plots following the treatments (p = 0.034) with the invaded plots differing from the adjacent uninvaded (p = 0.002). *Impatiens parviflora* is highly unlikely to impact native species richness of invaded communities, which may be associated with its limited ability to create a dense canopy, a modest root system or the fact the *I. parviflora* does not represent a novel and distinctive dominant to the invaded communities. Concerning its potential impacts on species composition, the presence of native clonal species (*Athyrium filix-femina*, *Dryopteris filix-mas, Fragaria moschata*, *Luzula luzuloides*, *Poa nemoralis*) on the adjacent uninvaded plots likely makes them different from the invaded plots. However, these competitive and strong species are more likely to prevent the invasion of *I. parviflora* on the adjacent uninvaded plots rather than being themselves eliminated from the invaded communities.

## Introduction

*Impatiens parviflora* is one of the most widespread aliens to Central Europe. Dense populations inhabit most of the sites available, *Impatiens parviflora* is sometimes even considered to have decreased in its occurrence in the last years and to be in “post-invasive stage”. Populations of *I. parviflora* have also been documented to be limited by the occurrence of parasites, such as *Puccinia komarovii* [1] and it ranks among the few aliens that have been documented to be attacked by a parasite from its native range in the invaded range [2]. However, this alien is often seen dominating herbal layer communities of invaded forests, including sites with relativelly low hemeroby, where it potentially could impact rare native species [3]. *Impatiens parviflora* was found to be the only alien from selected target species to dominate in more than three basic types of habitats [4]. Its ability to use light in a very efficient way has also been documented [5], explaining its tolerance towards low light conditions. Its annual rate of spread was estimated to be up to 24 km yr^−1^ on British Isles [6].

The invasion of *Impatiens parviflora* is not really likely to cause an on-going decline of native *Impatiens noli-tangere*, but the autecology of this alien and its interaction with other native species of invaded communities is surprisingly poorly documented [3]. Population densities of *I. parviflora* were found to negatively correlate with herbal layer diversity of invaded forests [7]. *Impatiens parviflora* invades disturbed forests with depauperated herbal layer communities most easily, while pristine forests with rich herbal layer communities represent an effective barrier against the invasion of this alien [7]. When comparing pristine forests, disturbed forests and forests dominated by either alien *I. parviflora* or native *Carex brizoides*, herbal layers of invaded commuties were found to resemble that of disturbed forests with lower diversity and a large share of hemerophilic species. The authors interpreted this phenomenon as being caused by the spread of either invasive alien *I. parviflora* or native *C. brizoides* [8]. Contrary to that, Lončáková and Mandák (unpublished data) found no changes in neither species diversity nor species composition associated with the invasion of *I. parviflora* in their removal field experiment.

It seems clear that the invasion of *I. parviflora* is associated with structural changes in herbal layers of invaded forests – mainly with diversity loss and spread of hemerophillic and ruderal species. These changes of the vegetation structure are likely to be associated with changes in site conditions – mainly with growing nutrient levels and disturbance. Both of these can be directly caused by humans or their pets, which is especially true in forests near large human settlements. However, humans may impact the site conditions for forest understorey species even indirectly. The atmospheric deposition of nitrogen is enhanced due to human activities, namely the spread of automobilism. Moreover, numbers of game animals are kept high in many forests of central Europe, partly due to the absence of large predators and partly due to feeding and keeping the animals for recreational hunting purposes. *Impatiens parviflora* is well documented to positivelly respond to the ruderalization tendencies [9], disturbance [10] and increasing nutrient levels [11].

It is difficult to judge to which degree the changes in vegetation of herbal layers are caused by the invasion of *I. parviflora* or if *I. parviflora* just profits from changes caused by another factors – is *I. parviflora* a driver or just a passenger of the ongoing changes? This paper aims to help answer this question by comparing heavily invaded vegetation with dominant *I. parviflora*, heavily invaded plots from which *I. parviflora* had been removed and non-invaded or minimally invaded plots with conditions suitable for the invasion of *I. parviflora*. The “removal” plots were assumed to reveal changes following the invader’s removal, while the adjacent uninvaded plots were set to reveal the long-term state of non-invaded stands. Using a combination of experimental and comparative approach, this case study aims to answer the following questions:

1. Does the invasion of *I. parviflora* affect native species richness and composition of invaded communities – will native species richness and composition change following the invader’s removal?
2. How do the invaded and removal plots differ from adjacent uninvaded or minimally invaded vegetation, representing conditions prior to the invasion by *I. parviflora*?

If the vegetation on removal plots develops in a different way compared to the invaded plots, it suggests that *I. parviflora* works as a factor affecting native diversity or species composition of invaded communities. In this case, *I. parviflora* would be the “driver” of the changes associated with the invasion. However, if the vegetation on removal and control invaded plots is the same at the end of the experiment, it shows that *I. parviflora* itself does not affect the vegetation and more likely acts as a “passenger” of the environmental changes associated with the invasion.

## Methods

### Areas Used for the Field Experiment and Experimental Plots

The experiment was set in two areas of central Czech Republic (Central Europe) – valley of Botič brook (around the town of Průhonice) and surroundings of the city of Kladno. Both of these regions contain mesophilous and semi-thermophilous deciduous forests with varying degree of degradation of the herbal layer community. The herbal layer community is very likely to be impacted by the presence of numerous game animal populations and human activities often resulting in the expansion of ruderal species, such as *Aegopodium podagraria* or *Geranium robertianum*. Besides forests, landscape of both of these areas consists of intensivelly used agricultural or urban areas. All the experimental plots were situated on a public land and the author was not obliged to have any permissions, since no plant material was collected besides the removal of the invasive alien *I. parviflora*.

The experimental plots were set in deciduous or mixed forests with varying degree of disturbance and hemeroby. Since it was not possible to locate plots randomly, they were spatially clustered in the following localities: Průhonice region; i) riparian and ruderalized forests with *Alnus glutinosa* and *Fraxinus excelsior* around the Botič stream; and ii) mesophilous forests with *Quercus robur* and *Q. petraea* in the Milíčov forest reserve. Kladno region: i) Kladno – Ostrovec suburb – a reserve with mesophilous forests with *Fagus sylvatica*; ii) Smečno – Kopaniny – mesophilous forest with *Quercus robur* and *Fagus sylvatica*; iii) Mount of Vinařice reserve – nutrient rich slope/scree forest with *Acer* sp.div. and wet ruderalized forest with *Populus tremula* iv) Šternberk and Hradečno villages – ruderalized forest with *Quercus robur* and *Larix decidua*; v) Libušín village – mesophilous forest with *Fagus sylvatica*. Within these localities, plots were located 10–100 meters apart from each other, while single localities were located 1–5 km apart. The two regions (Kladno and Průhonice) are 60 km apart from each other.

### Experimental Design

In late April 2008, 38 experimental plots were established in two areas in Central Bohemia. Random number generator was used to allocate treatments to experimental plots – *I. parviflora* was removed from half of the plots (19). Additional 19 plots with low abundance of *I. parviflora* (up to cca 10% of cover - termed “adjacent uninvaded”) were set at the time *I. parviflora* was being removed from selected plots, resulting in 57 plots altogether. Species on these adjacent uninvaded plots could have been expected to be recruited from the same species pools as on invaded plots and these sites could have been expected to have similar site conditions, due to being both spatially close and in the same type of vegetation.

Altogether, 57 plots were established and visited 5 times during each year. The present species were recorded and their cover (%) was estimated.

Design of the field experiment followed the Before-After-Control-Impact scheme and the interaction term between the treatment (removal of *I. parviflora*) and time (series of 5 visits during each year) was expected to reveal possible changes following the invader’s removal, indicating the impact of *I. parviflora* upon the forest floor community. The test on the “treatment” as a main factor was applied for the final sampling in September 2009 to reveal differences among the three types of plots at the end of the experiment, resulting from different treatment levels.

The experiment was set up in late April 2008 and was run till September 2009. In 2008, the experimental plots were established in dense stands of *I. parviflora* seedlings, where the most severe impact upon herbal community could have been presumed. On the other hand, dense stands of *I. parviflora*’s seedlings could have impacted the herbal layer community even on removal plots (before the seedlings of *I. parviflora* were removed), and such an impact would not have been detected during the season 2008. To account for possible invader’s impact in early phases of the vegetation season (e. g. competition between seedlings of *I. parviflora* and native species), *I. parviflora*’s seedlings were removed early enough not to impact other species on removal plots of this case study in 2009.

### Data Analysis

The data were analyzed using a hierarchical generalized mixed effect models [12], which made it possible to deal both with the autocorrelation within the data and the non-normal error structure properly. The factors “region” (Průhonice and Kladno region), “locality” (nested in region) and “plot identity” (nested in “locality”) were considered random, while “time” factor (nested in “plot identity”), representing consecutive samplings through the year, “year” (2008 and 2009) and “treatment” (removal, invaded and adjacent uninvaded plots) were considered fixed factors. The 2 - way interaction between time (representing consecutive samplings within a year) and treatment was of the most interest, since it would reveal possible differences in the development of plots within each season. Further, a three way interaction between the fixed factors treatment, time and year would show if the seasonal development of plots differed according to the treatments and the two years of the experiment’s duration. Numbers of native herbal layer species in each plot (including tree and shrub seedlings) recorded through the seasons 2008 and 2009 were the importance values of the response variable. The distribution related assumptions about the data were tested using a Shapiro-Wilk normality tests. Therefore, a poisson error structure was assumed to be more appropriate than normally distributed, given the generally low numbers of species found in plots with some of those being actually empty and not harbouring any native species. All univariate analyses were processed in “R” [13].

Another hierarchical mixed effect model with poisson error structure [12] was used to test differences in numbers of native species found in the invaded, removal and adjacent uninvaded plots with low cover of *I. parviflora* at the end of the experiment. The treatment was the explanatory variable, while “region” and “locality” (nested in region) were set as random factors. In both generalized mixed effect models, the significance of particular terms was tested via deletion tests, when the growth of unexplained deviance following removal of a particular term was tested using a Chi^2^-test.

Possible differences in species composition following the removal of *I. parviflora* were tested using direct ordination models (CCA and RDA) and Monte – Carlo permutation tests [14]. An indirect model (DCA) was used to decide whether to use a linear or an unimodal approximation [14]. A split-plot design was used, with plot identities (whole-plots) being freely permuted and samplings within sites (split-plots) were not permuted [14]. The interaction term between treatments and time (samplings within plots) was used to test possible changes in plots following the removal of *I. parviflora*. Year, region, localities, time and plots’ identities were set as covariables, while the three interactions between sampling time and three levels of treatment were predictor variables. Since it does not really change the results, the time was considered a continuous variable in the ordination models, as recommended [15]. An ordination plot with three interaction terms (3 dummy variables representing the treatment levels x time as a continuous variable) allows for a much more straightforward interpretation.

Parallel to the univariate analysis of native species richness, treatment’s main effect at the end of the experiment was tested using the ordination models and Monte-Carlo permutation test as well. The three dummy variables representing three levels of the treatment (removal, invaded and adjacent uninvaded) were set as predictor variables, while regions and localities were covariables.

All multivariate ordination analyses were performed twice, once with estimated percentages of species’ covers to reveal possible differences in species’ abundances and once with binary data of presence and absence of species to reveal possible differences based on species composition only [16]. *Impatiens parviflora* was excluded from all the analyses.

The data is available in Data S1, which is included as a supplementary material.

## Results

18 of the original 19 control plots, 17 removal plots and 18 adjacent uninvaded or minimally invaded plots made it to the final sampling in early September 2009. In the autumn 2009, several plots were destroyed by wild pigs (*Sus scrofa*), so the field experiment had to be finished. Moreover, some of the plots “disappeared” during the season and were “rediscovered” during the last two sampling times. This concerned invaded plots mostly, since it was sometimes difficult to locate a particular plot precisely, without disturbing the stands of *I. parviflora*. It was considered a minor evil to have missing data during the season than to disturb the invaded plots. Moreover, such “hidden” plots were usually rediscovered during the later phases of the season, when the cover of *I. parviflora* had decreased substantially.

Together, 50 species were recorded on the invaded plots, while 65 species were recorded on plots from which *I. parviflora* had been removed and 57 species were found on adjacent uninvaded plots with low abundances of *I. parviflora*. The numbers of native species recorded in plots (Table 1) did not reveal significantly different dynamics following the removal of *I. parviflora* (Chi^2^ = 7.229, DF = 8, p = 0.5121), nor was the treatment’s main effect significant when it was tested at the end of the experiment (Chi^2^ = 0.4757, DF = 2, p = 0.7883). However, the interaction between the time (representing the consecutive samplings throught both seasons) and year (seasons 2008 and 2009) was significant (p = 0.037), showing that species richness found in plots revealed different dynamics throught the two years of the experiment, however, this was independent on the treatment levels – Table 2.

**Table 1:** Mean numbers and standard deviations of native herbal species recorded during the experiment.

year	time of sampling	invaded plots	mean cover of I. parviflora	removal plots	uninvaded plots
2008	last week of April	4.05±1.99	81.05	4.05±2.95	4.53±2.46
2008	first week of June	3.58±1.80	80.00	4.63±3.52	4.16±2.27
2008	second week of July	3.47±1.35	47.53	4.58±3.37	4.16±2.09
2008	last week of August	3.68±1.53	9.68	4.79±3.54	3.95±2.32
2008	last week of September	3.95±1.93	0.16	4.05±2.80	3.63±2.43
2009	first week of April	1.27±1.16	15.50	1.19±1.17	1.78±1.64
2009	second week of May	3.65±3.05	63.33	3.61±3.04	3.74±2.68
2009	last week of June	3.84±3.03	49.71	3.76±3.03	3.79±2.74
2009	first week of August	4.11±3.13	24.47	4.03±3.12	4.32±3.13
2009	second week of September	3.81±2.74	0.22	3.74±2.74	3.90±2.51

The table also shows mean cover of *I. parviflora* on invaded plots, as recored during each of the 5 consecutive sampling times within each season.

**Table 2:** Analysis of deviance table of hierarchical generalized mixed-effect models applied to the data.

tested term	deviance inflation	Chisq	DF	p - value
main effect of treatment	0.9	0.9653	2	0.6172
main effect of time	2.2	2.2898	4	0.6826
main effect of year	0.2	0.2728	1	0.6015
treatment:time interaction	7.2	7.229	8	0.5121
treatment:year interaction	1.9	1.8602	2	0.3945
time:year interaction	10.2	10.191	4	0.03733
3 - way interaction (time:year:treatment)	1.9	1.9013	8	0.9839

Maximal model (with all possible main effects and interactions of fixed factors) was simplified and less complex models were created. First, a three-way interaction between treatment, time and year was removed, followed by two-way interactions and then main effects of treatment, time and year. The table shows the growth of residual deviance associated with omitting each term or interaction, which was tested using Chi-square tests.

Total cover of *Impatiens parviflora* on invaded plots varied considerably through the season (Table 1). In 2008, the largest covers (presumably correlating with biomass) were detected during the first and second sampling times (end of April, beginning of June). In 2009, the plots were sampled and treated earlier (starting in mid April) to avoid possible impact of *I. parviflora*’s seedlings and the largest cover of *I. parviflora* was recorded during the second and third sampling time (end of May, beginning of July). In 2009, the mean covers of *I. parviflora* were lower, however, both in 2008 and 2009, the cover of *I. parviflora* sharply declined through the season, decreasing almost to zero values recorded during the last sampling time (mid September in 2008 and beginning of September in 2009).

The ordination analysis, performed on the species’ covers data, revealed marginally significant differences in the development of plots through the experiment (p = 0.052) and significant differences when the binary presence-absence data were used (p = 0.032). Of the pairwise combinations of treatment levels, the invaded plots differed marginally significant from removal plots (p = 0.072) and significantly from the adjacent uninvaded plots (p = 0.002), when using the species’ covers data. When using the binary presence – absence data, the invaded plots differed significantly from the adjacent uninvaded (p = 0.004).

When testing the treatment’s main effect at the end of the experiment, the species’ covers gave a marginally significant result in the overall test with all the treatment levels (p = 0.07), with the adjacent uninvaded plots differing significantly from the removal plots (p = 0.034) and invaded plots (p = 0.004). The binary presence – absence data gave marginally significant result only when comparing the invaded and adjacent uninvaded plots (p = 0.05– table 3).

**Table 3:** Results of direct gradient ordination analyses performed on the data.

Predictors	data	F-ratio	p-value	Trace
treatment × sampling time interaction	species’ covers (CCA)	1.739	0.052	0.029
treatment × sampling time interaction (removal vs. invaded plots)	species’ covers (CCA)	2.417	0.074	0.024
treatment × sampling time interaction (removal vs. adjacent uninvaded plots)	species’ covers (CCA)	1,45	0.368	0.017
treatment × sampling time interaction (invaded vs. adjacent uninvaded plots)	species’ covers (CCA)	2.208	0.002	0.027
treatment × sampling time interaction	binnary data (RDA)	1.523	0.034	0.002
treatment × sampling time interaction (removal vs. invaded plots)	binnary data (RDA)	1.452	0.116	0.002
treatment × sampling time interaction (removal vs. adjacent uninvaded plots)	binnary data (RDA)	0.772	0.9380	0.001
treatment × sampling time interaction (invaded vs. adjacent uninvaded plots)	binnary data (RDA)	2.386	0.004	0.003
treatment (main effect)	species’ covers (CCA)	1.375	0.07	0.309
treatment (main effect - removal vs. invaded plots)	species’ covers (CCA)	1.077	0.370	0.135
treatment (main effect - removal adjacent uninvaded plots)	species’ covers (CCA)	1.740	0.034	0.260
treatment (main effect - invaded vs. adjacent uninvaded plots)	species’ covers (CCA)	1.941	0.004	0.304
treatment (main effect)	binnary data (RDA)	1.139	0.2280	0.034
treatment (main effect - removal vs. invaded plots)	binnary data (RDA)	1.201	0.2360	0.027
treatment (main effect - removal vs. adjacent uninvaded plots)	binnary data (RDA)	0.824	0.742	0.019
treatment (main effect - invaded vs. adjacent uninvaded plots)	binnary data (RDA)	1.523	0.05	0.032

For each of the analyses, the table shows the tested term (main effect or interaction), type of data (species’ covers estimates or binary presence – absence), results of test statistics (F-ratio and p-value) and Trace. Trace is, in this case, a sum of canonical eigenvalues of the model and represents the ordination model’s explanatory power.

## Discussion

The mean numbers of species found in plots revealed only miniscule differences among the treatments, be it through the experiment or at the end of it, at the last sampling in September 2009. This shows that i) removal of the alien *I. parviflora* did not stimulate the increase or change of native species richness and ii) native species richness did not differ between invaded plots with large covers of *I. parviflora* and adjacent uninvaded or little invaded plots with comparable site conditions.

Therefore, the invasive alien *I. parviflora* seems to have only a miniscule impact on native species richness of invaded forests’ herbal layer. This is supported not only by non-significant results of the tests, but mainly by comparable native species richness on the three types of plots through and at the end of the experiment (Table 1). The low (if any) impacts on native species richness can be caused by several factors:

- Although *I. parviflora* is a widespread alien which can be locally very abundant and often creates dense stands on small spatial scales, the plant is subtle and does not really create very dense canopy, as compared to other invasive aliens [17]. Moreover, being an anual species, it has a very modest root system and is very unlikely to compete with native species via belowground competition.
- Although *I. parviflora* tends to be very abundant in the beginning of the season, its cover decreases sharply after flowering and is generally low since early July, which may decrease its impact on native species. The decrease in the cover of *I. parviflora* does not need to be related to neither changes in site conditions nor to the competition by other species. It seems to be driven simply by the phenology of the invasive annual *I. parviflora*’s populations, since there is no need for an annual plant to persist long after blooming and seeding.
- *Impatiens parviflora* most usually competes with forest herbs and saplings of tree species, and both of these can be presumed to be well adapted to low light conditions. Intensive competition during the early phases of season seems to be the only mechanism, how *I. parviflora* could impact native species diversity. It could compete with heliophilous early spring forest species, such as *Ficaria verna*, *Hepatica nobilis* or *Anemone nemorosa*, but nothing like this has been observed during this field experiment. In this way, the character of invaded communities and traits of particular native species (adaptation to low light conditions) can co-determine the low impact of this invasive alien, acting in concert with traits of the invader [16], [17].

**Figure 1:**
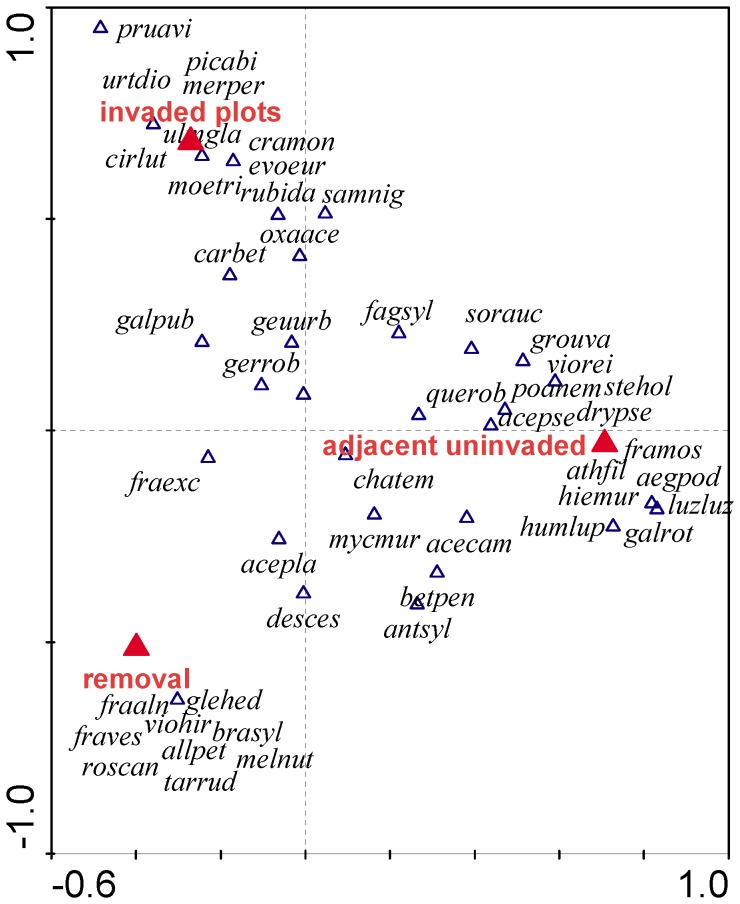
Ordination plot of a model testing the final state of the plots according to the original treatments: invaded, removal of *I. parviflora* and adjacent uninvaded or minimally invaded plots. The species’ percentage cover estimates were used as importance values. The adjacent uninvaded plots were dominated by native clonal perennials (*Aegopodium podagraria*, *Athyrium filix femina*, *Dryopteris pseudomas*, *Fragaria moschata*, *Luzula luzuloides*, *Poa nemoralis*). However, the model with all three treatment levels was only marginally significant (p = 0.07) and it was the adjacent uninvaded plots that differed from the invaded plots both through and at the end of the experiment – see Table 3. The first cannonical axis explains 3.7% of the variablity in the data, the second cannonical axis explains 1.5%. The figure shows all species recorded in the removal, invaded and adjacent uninvaded plots during the last sampling time in early September 2009. Abbreviations: acecam  =  *Acer campestre*, acepla  =  *Acer platanoides*, acepse  =  *Acer pseudoplatanus*, aegpod  =  *Aegopodium podagraria*, allpet  =  *Alliaria petiolata*, antsyl  =  *Anthriscus sylvestris*, athfil  =  *Athyrium filix-femina*, betpen  =  *Betula pendula*, brasyl  =  *Brachypodium sylvaticum*, carbet  =  *Carpinus betulus*, chatem  =  *Chaerophyllum temulum*, cirlut  =  *Circaea lutetiana*, cramon  =  *Crataegus monogyna*, desces  =  *Deschampsia cespitosa*, drypse  =  *Dryopteris pseudomas*, evoeur  =  *Evonymus europaea*, fagsyl  =  *Fagus sylvatica*, framos  =  *Fragaria moschata*, fraves  =  *Fragaria vesca*, fraaln  =  *Frangula alnus*, fraexc  =  *Fraxinus excelsior*, galpub  =  *Galeopsis pubescens*, galrot  =  *Galium rotundifolium*, gerrob  =  *Geranium robertianum*, geuurb  =  *Geum urbanum*, glehed  =  *Glechoma hederacea*, grouva  =  *Grossularia uva-crispa*, hiemur  =  *Hieracium murorum*, humlup  =  *Humulus lupulus*, luzluz  =  *Luzula luzuloides*, melnut  =  *Melica nutans*, merper  =  *Mercurialis perennis*, moetri  =  *Moehringia trinervia*, mycmur  =  *Mycelis muralis*, oxaace  =  *Oxalis acetosella*, picabi  =  *Picea abies*, poanem  =  *Poa nemoralis*, pruavi  =  *Prunus avium*, querob  =  *Quercus robur*, roscan  =  *Rosa canina* agg., rubida  =  *Rubus idaeus*, samnig  =  *Sambucus nigra*, sorauc  =  *Sorbus aucuparia*, stehol  =  *Stellaria holostea*, tarrud  =  *Taraxacum* sec. *Ruderalia*, ulmgla  =  *Ulmus glabra*, urtdio  =  *Urtica dioica*, viohir  =  *Viola hirta*, viorei  =  *Viola reichenbachiana.*

Contrary to native species richness, plots revealed different dynamics of species composition following the treatments, provided binary presence – absence data were used as importance values. This suggests that the differences were actually caused by the presence or absence of specific species rather than just changes in species’ abundances. However, when considering the pairwise tests of single treatment levels both through and at the end of the experiment, it was the adjacent uninvaded plots that differed from the invaded plots, both when the species’ covers or binary presence-absence data were used for the ordination analyses. Fig. 1 shows that the adjacent uninvaded plots were dominated by native clonal species, such as *Aegopodium podagraria*, *Athyrium filix-femina*, *Dryopteris filix-mas*, *Fragaria moschata*, *Luzula luzuloides* and *Poa nemoralis*. These species may work as a barrier against the invasion of *I. parviflora* [7] rather than being eliminated from the invaded communities themselves. Native species like *Ficaria verna*, *Carpinus betulus*’s seedlings, *Vaccinium myrtillus*, *Pteridium aquilinum* and tuft grasses were found to have locally strong inhibitory effect on the growth of *Impatiens parviflora* [18]. This mechanism may also explain why the adjacent uninvaded plots have not been invaded massively so far, even though the site conditions are surely suitable for the growth of *I. parviflora*, which is known to be able to colonize a range of habitats with varying site conditions [4], [18], [19]. More likely, slight shifts in site conditions (e. g. lower trophic levels or less ruderalization) or just stochastic factors favour competitively strong native species, so *I. parviflora* is not able to gain dominance over such stands. Uninvaded plots might slightly differ in site conditions from the invaded plots, but they can still be assumed to reveal the state of the uninvaded community, with native species preventing *I. parviflora* from acquiring dominance.

*Impatiens parviflora* does not appear to be the only alien with miniscule if any impacts on diversity and composition of invaded communities. *Mimulus guttatus* was found to be too weak dominant to impact native species [16], [17]. On the other hand, even some dominant alien species, such as *Impatiens glandulifera* or *Helianthus tuberosus*, were found to have a low community-level impact. It was assumed that these species usurp the dominance from native dominants without actually changing the site conditions dramatically [16], [17]. Alien species that represent new and distinctive dominants to communities that had been lacking distinctive dominant species prior to the invasion, tend to have the highest impact upon the diversity and composition of native species. This effect was observed in case of such aliens as *Fallopia* sp. div. or *Heracleum mantegazzianum*. In this way, characteristics of the invaded community, such as presence of native dominant species, co-determine the magnitude of the alien’s impact. The character of the invaded community may also co-determine the low community-level impacts of *I. parviflora*. The vegetation in the invaded communities appears to be limited by other factors – mainly light availability. *Impatiens parviflora* certainly does not appear to be a distinctive dominant compared to native species like *Aegopodium podagraria* or *Luzula luzuloides*. Similar types of communities, deciduous forests classified as *Potentillo albae – Quercetum*, have been reported to reveal a loss of diversity following an invasion of a tree species *Carpinus betulus* [20].

The invasion by *I. parviflora* is confounded with degradation of forest floor communities, associated with nitrification by numerous game animals and often leading to the expansion of ruderal species, such as *Geranium robertianum*, *Chelidonium majus*, *Galeopsis pubescens* and especially *Aegopodium podagraria*, and the invasion may impact resident communities more when acting in concert with these factors [8], [21]. A strongly positive response of *I. parviflora* to nutrient addition was documented [22]. On the other hand, *I. parviflora* has been observed to avoid patches with high biomass of native species and to colonize empty spaces as an addtitional element, at least in well preserved forests [23]. Although *I. parviflora* is known to be able to thrive and even gain dominance in a relativelly wide range of environments [24], its populations may still be sensitive to variations in climate and other environmental factors. For example, *I. parviflora* is known to be sensitive towards drought periods. In exceptionally dry years, many of the long-term occupied sites may become unfavorable for the growth of this alien species, or *I. parviflora* may no longer be able to compete with native species successfuly. From a long-term perspective, these factors may cause the populations of *I. parviflora* to be patchy rather than continuous homogenous stands and this effect is likely to be apparent even on the level of microsites. On the contrary, [25] reported that *I. parviflora* remained a long-term dominant plant species in the study area in Hungary.

Spatial distribution of localities with experimental plots did not exactly correspond to the types of habitats. Therefore, the blocking based on the localities reflected the spatial autocorrelation of plots rather than being completely homogenous in terms of habitats. On the other hand, experimental plots within localities did not reveal high diversity among plots and shared many dominant native species, such as *Aegopodium podagraria*, *Geranium robertianum* and *Galeopsis pubescens*. For these reasons, it is highly unlikely that similarities or differences among experimental plots would bias the results.

Plot size is another issue possibly affecting the results. Small scale plots (1×1 m) were chosen, as it would not have been possible to eradicate *I. parviflora* from larger plots without inducing severe disturbance to the removal plots. Given the low density of the understorey.

species’ populations, most plots harboured just 5–7 species with some of the plots actually being empty and not harbouring any native species. Vegetation on such small scale plots is actually prone to be influenced by edge effects. Removal plots could have been impacted by the surrounding stands of *I. parviflora*, while, conversely, the vegetation on invaded plots could have been influenced by the *I. parvilora*’s being trampled in the surroundings when recording the vegetation data. To minimize the edge effects on the removal plots, *I. parviflora* was destroyed also in the 0.2–0.3 m belt around the removal plots. On the contrary, to minimize the edge effects on the invaded plots with intact stands of *I. parviflora*, plots were inspected and data collected from a distance to leave a 0.2–0.3 m wide belt of intact vegetation with *I. parviflora* around the invaded plots.

All the results need to be interpreted with certain caution, the length of the field experiment being its main limitation. Removal plots may need more time than just two vegetation seasons to get close to their pre-invasion state because re-colonization by native species may procceed slowly. Thus, the question arises, how long should such an experiment run to provide valid results? If *I. parviflora* had been suppressing other species in the invaded community, removal plots would have been probably quickly colonized by hemerophilic species, a lot of which were present both in the experimental plots and in their vicinity. At the same time, better performance of tree seedlings could have been expected, especially during the second year (2009), since removal plots could not have been impacted by the high density of *I. parviflora* seedlings, observed in the early phases of the vegetation season in 2008. No such development has been observed. *Impatiens parviflora* is a an ephemerous plant and it is not really likely that its impacts upon the resident community would persist long after its removal. However, this does not really imply that the community develops quickly after the potential effect of *I. parviflora* has been ceased.

Even though this study adopts a combination of experimental and comparative approaches, the interpretations still have constraints due to spatial distribution and size of experimental plots as well as due to the limited time scale of the experiment. However, almost equal numbers of native species richness both through and at the end of the experiment suggest that *I. parviflora* does not suppress native vegetation. More likely, native species with dominant tendencies work as an effective barrier against its invasion, suggesting that *I. parviflora* is more likely a passenger of the ongoing changes rather than a driver of the degradation of invaded vegetation.

## Supporting Information

Data S1 **The file presents the input data for ordination models, which tested the possible differences in species composition and also univariate mixed-effect models, which tested possible differences in species richness.**
(XLS)
